# Imparting Stability to Chiral Helical Gold Nanoparticle Superstructures

**DOI:** 10.1021/acs.langmuir.4c01531

**Published:** 2024-07-17

**Authors:** Yicheng Zhou, Yuyu Zhang, Nathaniel L. Rosi

**Affiliations:** † Department of Chemistry, University of Pittsburgh, Pittsburgh, Pennsylvania 15260, United States; ‡ Department of Chemical and Petroleum Engineering, University of Pittsburgh, Pittsburgh, Pennsylvania 15260, United States

## Abstract

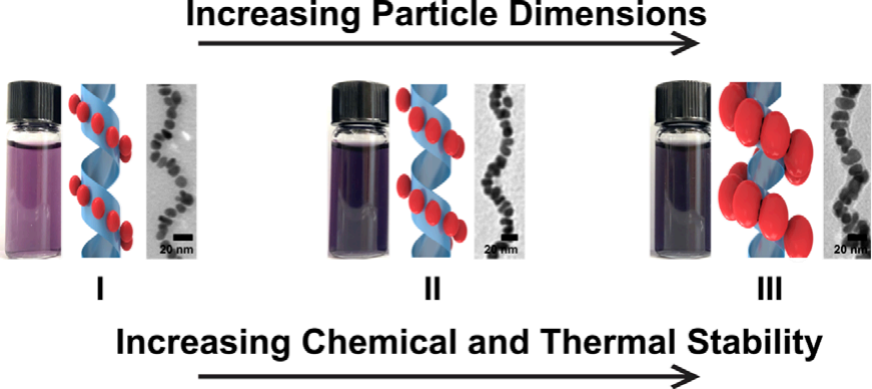

Realizing the promise of chiral inorganic nanomaterials hinges on improving their structural stability under various chemical and environmental conditions. Here, we examine the stability of 1-D gold nanoparticle (Au NP) single helices prepared using the amphiphilic peptide conjugate C_*x*_-(PEP_Au_^M-ox^)_2_ (PEP_Au_^M-ox^ = AYSSGAPPM^ox^PPF; *x* = 16–22). We present a general template-independent strategy of tuning helix stability that relies on controlling the dimensions of constituent NPs. As NP dimensions increase, Au NP single helices become both more thermally stable and more stable in the presence of chemical denaturants and protein digestion agents (e.g., urea and proteinase K, respectively). We use this strategy for imparting helix stability to create colloidal suspensions of thermally robust Au NP single helices which maintain their plasmonic chiroptical activity up to ∼80 °C.

## Introduction

Chiral nanoparticle (NP) superstructures have attracted broad interest across multiple research communities due to (i) their inherent structural complexity, which motivates continued development of sophisticated and versatile NP assembly methods, and (ii) their unique chiroptical properties, which enable the creation of new sensors and optical metamaterials.^1−5^ These materials are typically fabricated by assembling NPs using chiral molecular templates (e.g., peptide fibers,^6−8^ nucleic acid assemblies,^5,9−11^ polymers and liquid crystals^12−15^). Though numerous studies have detailed their assembly, structure, and properties, less attention has been devoted to studying their structural stability under the various chemical and environmental conditions relevant to their constitution. Realizing strategies for tuning and improving stability represent critical milestones in the continued development of this class of materials and underpins their future use as functional components in devices and composite materials.

The templates used for constructing chiral NP superstructures are often based on intermolecular interactions which can be sensitive to different environmental conditions. Because each templating species may require different conditions for retaining chiral structure, it is important to identify general template-independent strategies for imparting stability. To this end, postsynthetically embedding superstructures in a matrix (e.g., silica^16^) may be used to fix particles in place and prevent disassembly. However, such stabilizing matrices may limit access to and chemical manipulation of the NP, significantly limiting control over interfacial interactions that affect optical properties and compatibility with other materials. Varying the dimensions of constituent NPs may present a general and template-independent means of controlling structural stability. Here, we study the stability of single-helical NP superstructures as a function of NP dimensions and demonstrate that stability increases as NP dimensions increase. We leverage this strategy for increasing helix stability to create colloidal suspensions of structurally robust Au NP single helices that exhibit pronounced and stable plasmonic chiroptical activity.

## Experimental Section

### General Methods and Materials

All chemicals were purchased from commercial suppliers and used as received unless otherwise specified. Nanopure water (18.2 MΩ-cm) was obtained using a Barnstead Diamond water purification system. Peptide syntheses were carried out on a CEM MARS 6 synthesis microwave reactor. Peptides and peptide conjugates were purified using an Agilent 1200 reverse-phase high-performance liquid chromatography (RP-HPLC) system equipped with multiple wavelength detectors and a Zorbax-300SB C_18_ column. A linear gradient of a binary solvent system (A: 0.1% formic acid in nanopure water; B: 0.05% formic acid in acetonitrile), ramping from 95% buffer A and 5% buffer B to 5% buffer A and 95% buffer B over 30 min, was used to elute samples. The molecular masses of purified peptides and peptide conjugates were confirmed using a Shimadzu liquid chromatography–mass spectroscopy (LC-MS) 2020 system using a direct injection method with an electron spray ionization (ESI) probe in positive and negative scan mode over a total run of 6 min. An Agilent 8453 UV–vis spectrometer with a quartz cuvette (10 mm path length) was used to quantify peptide conjugate based on tyrosine (1280 M^–1^ cm^–1^) absorbance at 280 nm. All microscopy measurements were performed using ImageJ (NIH, USA) software.^17^

### Fmoc Solid-Phase Peptide Synthesis

Peptides were synthesized by manual microwave-assisted Fmoc (Fmoc = fluorenylmethyloxycarbonyl) solid-phase methods using a CEM MARS 6 microwave and NovaSyn TGA Fmoc-Phe resin (Millipore, Burlington, MA, USA). The resin was first soaked in dimethylformamide (DMF) for 30 min, and a 2 mL Fmoc-deprotection solution consisting of 20% 4-methylpiperidine in DMF was added. Fmoc deprotections were performed with a 1 min ramp to 75 °C, followed by a 2 min hold at that temperature with stirring. Thereafter, excess solution was drained using a filtration manifold and washed with 2 mL of DMF (3×). Thereafter, a 1.25 mL coupling solution consisting of 0.1 M HCTU (*O*-(1*H*-6-chlorobenzotriazole-1-yl)-1,1,3,3-tetramethyluronium hexafluorophosphate, 5 equiv to resin) in NMP (1-methyl-2-pyrrolidinone), vortexed with Fmoc-protected amino acid (5 equiv to resin) and DIEA (*N*,*N*-diisopropylethylamine, 7 equiv to resin), was added. Coupling reactions were then performed with a 1 min ramp to 75 °C, followed by a 5 min hold at that temperature while stirring. Thereafter, excess reagent was drained and the residue was washed with DMF (3×). This coupling–deprotection cycle was repeated for every amino acid. Proline and adjacent amino acids were coupled twice to ensure complete reaction of the secondary amide group. The N-terminus was capped with 5-azido pentanoic acid (4 equiv to resin). The resin was then washed with DMF (3×), methylene chloride (3×), and methanol (3×). The peptides were then cleaved from resin using a 2 mL cleavage cocktail (95/2.5/2.5 trifluoroacetic acid/triisopropylsilane/H_2_O). Crude peptide was precipitated by adding cold diethyl ether (Et_2_O) and centrifuged to decant the supernatant. The peptides were then dissolved in 1/1 (v/v) acetonitrile/water and lyophilized. Solid peptide was stored at −20 °C.

### Preparation of Peptides and Peptide Conjugates

Details of oxidation of N_3_-PEP_Au_, syntheses of C_16_-dialkyne and C_18_-dialkyne, and copper-assisted “click” coupling of C_16_-dialkyne/C_18_-dialkyne to N_3_-PEP_Au_^M-ox^ to yield C_16_-(PEP_Au_^M-ox^)_2_, C_18_-(PEP_Au_^M-ox^)_2_, and modulator C_16_-(AYSSGA)_2_ were described in previous publications.^18,19^

### Synthesis of Au NP Single Helices “**I**”

**I** was prepared using established methods with minor modifications.^18,19^ Briefly, 20 nmol of lyophilized C_18_-(PEP_Au_^M-ox^)_2_ was dissolved in 200 μL 0.1 M HEPES (4-(2-hydroxyethyl)-1-piperazineethanesulfonic acid) buffer. Next, the sample was sonicated for 5 min, before it was left undisturbed at room temperature for 25 min. Toward the end of the 25 min incubation, a gold precursor solution was freshly prepared by vortexing 1:1 (v/v) 0.1 M gold(III) chloride trihydrate (HAuCl_4_) in H_2_O and 1 M triethylammonium acetate (TEAA) buffer for 1 min. After the 25 min incubation, 2 μL of the gold precursor solution was added to the peptide conjugate solution. Upon observing a black precipitate, the reaction vial was vortexed and left at room temperature for 18 h. The sample was then centrifuged (5 krpm, 10 min) and redispersed in 200 μL of 0.1 M HEPES buffer.

### Synthesis of Au NP Single Helices “**II**” and “**III**”

**II** was prepared following a previously reported procedure with slight modification.^20^ First, Au NP helices **I** were synthesized using the method described in the previous section, where the 200 μL of 0.1 M HEPES was replaced with 200 μL of 0.08 M HEPES/0.02 M citrate buffer. After sitting at room temperature for 18 h, a secondary gold deposition was initiated. A 1 μL amount of 0.1 M HAuCl_4_ was added to the solution, and then the vial was vortexed for 5 s. The sample was allowed to sit for 1 min; then 100 μL of 0.1 M hydroquinone, 0.08 M HEPES, and 0.02 M citrate buffer was added. The mixture was vortexed for 3 min, after which the color became dark purple; a precipitate was also observed. After 1 h, the solution was centrifuged (5 krpm, 10 min) and redispersed in 200 μL of 0.1 M HEPES to yield **II**. For Au NP single helices **III**, an additional secondary gold deposition step was performed on **II**. The resulting Au NP single helices were centrifuged and redispersed in 200 μL of 0.1 M HEPES to yield **III**.

### Synthesis of a Colloidal Suspension of Au NP Single Helices

A colloidal suspension of Au NP single helices was prepared using a previously reported synthesis^21^ with slight modification as described above in the syntheses of **II** and **III**. Briefly, a 10:20 nmol mixture of C_16_-(PEP_Au_^M-ox^)_2_ and C_16_-(AYSSGA)_2_ was dissolved in 250 μL of 0.08 M HEPES/0.02 M citrate buffer and sonicated for 5 min; then, 2.5 μL of 0.1 M CaCl_2_ was added. After ∼1 min incubation at room temperature, 1.5 μL of 1:1 (v/v) 0.1 M HAuCl_4_/1.0 M TEAA gold precursor solution was added. A black precipitate appeared after ∼2 s. Thereafter, the solution was vortexed for 30 s and then left on the bench for ∼16 h. The same vial was then subjected to a round of secondary gold deposition. A 1.5 μL amount of 0.1 M HAuCl_4_ was added to the vial, which was then vortexed for 6 s. After incubating for 1 min, 100 μL of 0.08 M HEPES, 0.02 M citrate buffer, and 0.1 M hydroquinone was added, the vial was vortexed for 3 min, and it was then incubated at room temperature for ∼16 h. Thereafter, the vial was centrifuged 2× at 6500 rpm for 15 min and redispersed in 300 μL of 0.1 M HEPES. The resulting suspension was sonicated for at least 30 min. We note that single helix syntheses based on C_16_-(PEP_Au_^M-ox^)_2_ and C_18_-(PEP_Au_^M-ox^)_2_ are virtually interchangeable, with the only difference being that the product helices have different average pitch values.^19^

### Transmission Electron Microscopy

TEM imaging was performed either on a FEI Morgagni 268 operated at 80 kV and equipped with an Advanced Microscopy Techniques (AMT) side mount charge-coupled device (CCD) camera system or on a Hitachi H-9500 microscope equipped with a Gatan OneView camera analyzed by Digital Micrograph software operating at 300 kV. For TEM images collected for colloidal suspensions of Au NP single helices, previously reported protocols were used.^18,19^ The remaining TEM samples were prepared by drop-casting 5 μL of sample onto a 3 mm diameter copper grid with Formvar coating. After 5 min, excess solution was removed by filter paper and the sample was air-dried for 1 min. A 5 μL amount of H_2_O was applied to the grid to remove excess solvent. After 1 min, the H_2_O was removed and the grid was allowed to dry in air for 5 min. Negatively stained TEM samples were prepared by adding 5 μL of 2% uranyl acetate solution to the grid after it was air-dried for 5 min. Next, after 5 min, the excess staining solution was removed by filter paper and the grid was allowed to dry in air for 5 min.

### Circular Dichroism Spectroscopy

Circular dichroism studies were performed on an Olis DSM 17 CD spectrometer using a quartz cuvette (1 mm path length) at 25 °C with a 10 nm/min scan rate and 5 nm bandwidth. For secondary structure studies, peptide conjugates were dissolved in 0.01 M HEPES, and spectra were collected from 190 to 250 nm. For plasmonic studies, spectra were collected from 450 to 800 nm. Under elevated temperature conditions, samples were heated at 5 °C increments from 25 to 90 °C with 5 min incubation at each temperature increment. For proteinase K (ProK) studies, the experiments were performed at 37 °C. For urea studies, the experiments were performed at room temperature.

### Thioflavin T (ThT) Fluorescence Assay

ThT fluorescence spectra were recorded on a Horiba Jobin Yvon (HJY) Fluoromax-3 spectrofluorometer using a quartz cuvette. Samples of either assembled fibers or single helices were incubated with ThT to a working concentration of 5 μM. They were excited at 450 nm, and emission was recorded from 460 to 600 nm. For ProK studies, spectra were collected at 37 °C. For elevated temperature studies, the cuvette was incubated for 5 min at desired temperatures and then spectra were collected.

### Stability Studies

Thermal studies were conducted by heating samples (peptide fibers or single helices) at 5 °C increments from 25 to 90 °C with 5 min incubation at each temperature increment. For urea studies, appropriate amounts of a urea stock solution in nanopure water were added at room temperature to samples of fully assembled peptide fibers or single helices to achieve final desired urea concentrations ranging from 0.005 M to 10 M. For ProK studies, appropriate amounts of a ProK stock solution in nanopure water were added to samples of fully assembled peptide fibers or single helices to achieve final desired ProK concentrations ranging from 0.02 mg/mL to 0.8 mg/mL. The resulting samples were incubated at 37 °C.

## Results and Discussion

**Figure 1:**
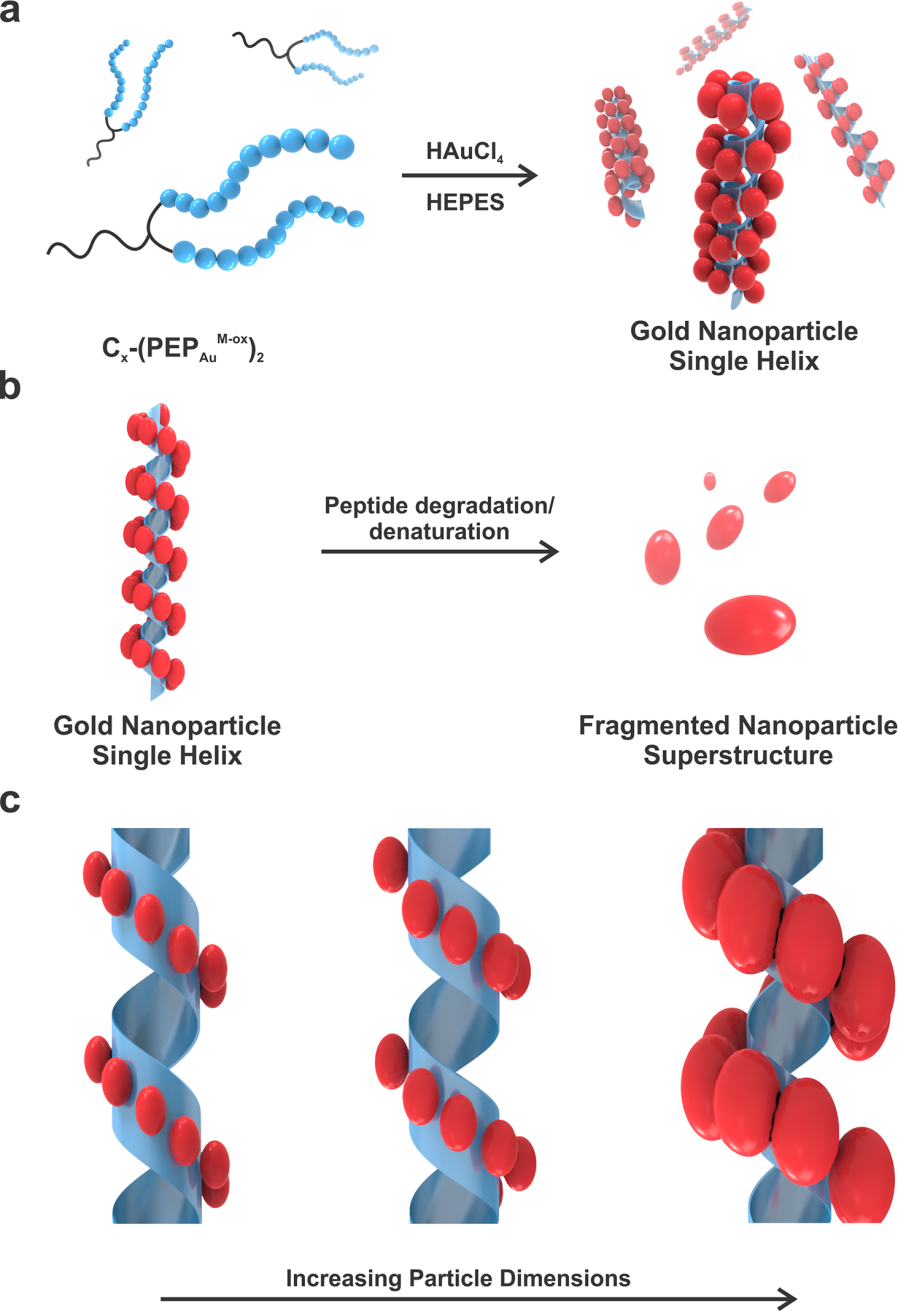
a) C_*x*_-(PEP_Au_^M-ox^)_2_ directs the formation of Au NP single helices in the presence of HAuCl_4_ and HEPES buffer. b) Degradation or denaturation of the peptide-based template leads to fragmentation of the helical Au NP superstructure. c) Au NP dimensions are controllable parameters that may affect single-helix stability.

Peptides and peptide-based molecules are versatile building blocks for constructing soft matter^22−27^ and directing NP assembly.^2,28−34^ We have developed versatile peptide-based strategies for assembling NPs into various structurally complex NP superstructures that utilize amphiphilic peptide conjugate molecules designed to both bind NP surfaces and assemble in aqueous media.^6,18,35,36^ The peptide is selected via *in vitro* evolution methods^37−39^ to adhere to a specific metal surface (e.g., Au, Ag, Pd). The C_*x*_-(PEP_Au_^M-ox^)_2_ peptide conjugate (C_*x*_, aliphatic tail where *x* = 16–22; PEP_Au_^M-ox^ = AYSSGAPPM^ox^PPF where M^ox^ = methionine sulfoxide) directs the formation of Au NP single helices^18,19^ when incubated in HEPES buffer with small amounts of HAuCl_4_.^38,40^ We previously determined that C_*x*_-(PEP_Au_^M-ox^)_2_ assembles into helical ribbons through a combination of hydrophobic interactions and β-sheet formation between the conjugates. Au NPs adhere to external faces of the ribbons to yield single helices (Figure 1a).^18^ Thus, the assembled helical ribbon serves as the underlying template which supports the single-helical structure. Degradation or denaturation of the ribbon via exposure to heat, enzymes, or chemical agents should result in fragmentation of the NP superstructure (Figure 1b).

**Figure 2:**
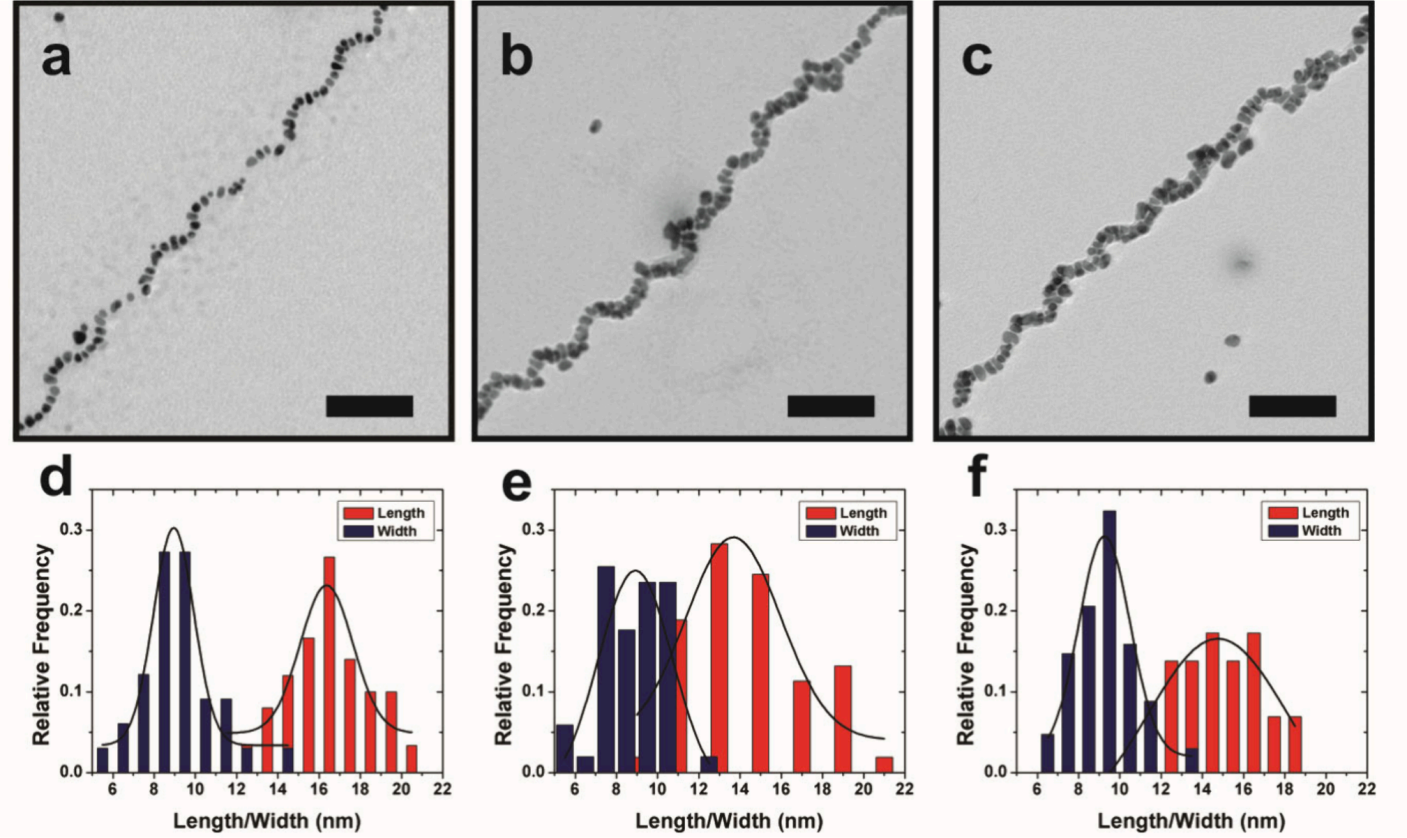
Characterization of Au NP single helices. TEM images (scale bar = 50 nm) and NP dimensions (based on at least 100 counts) for **I** (a, d), **II** (b, e), and **III** (c, f). The average NP lengths and widths increase (in nm) from **I** to **III**: length, **I** (13.5 ± 3.5), **II** (15.5 ± 4.1), **III** (17.9 ± 5.2); width, **I** (7.8 ± 2.4), **II** (9.5 ± 3.2), **III** (11.0 ± 3.7).

The size of constituent NPs (Figure 1c) is a controllable parameter that may affect single-helix stability. To study the effect of NP size on stability, we targeted a series of helices consisting of NPs of different dimensions (Figure 2). First, we used C_18_-(PEP_Au_^M-ox^)_2_ to prepare a sample of single helices (hereafter referred to as “**I**”).^18,19^ Product helices were purified via centrifugation to remove any free NPs and imaged using transmission electron microscopy (TEM) (Figure 2a). As reported previously, the NPs comprising the helices within **I** are oblong, and we can measure their lengths and widths (Figure 2d). By adapting previously reported procedures,^20^ we also prepared single-helix samples composed of progressively larger constituent NPs, **II** (Figure 2b,e) and **III** (Figure 2c,f), respectively. As dimensions increase, NPs merge closely together and the interparticle distances noticeably decrease (Figure 2a–c and Figure S1). UV–vis extinction spectra of the single-helix samples reveal a red shift and broadening of the plasmon resonance going from **I** to **III**, which is consistent with increasing NP dimensions and closely merged NPs (Figure S1). Having prepared **I**–**III**, we proceeded to study helix thermal stability as well as stability in the presence of a chemical denaturant (urea) and a peptide digestion agent (ProK).

### Thermal Studies

Since the peptide-based helical ribbons are held together through noncovalent intermolecular interactions, we elected to study helix stability as a function of temperature. From a practical perspective, it is important to determine a temperature range within which the helices maintain their structure and properties. We first examined the thermal stability of the peptide-based fibers that serve as the underlying template for the Au NP single helices. C_18_-(PEP_Au_^M-ox^)_2_ fiber samples in HEPES buffer were heated at 5 °C increments from 25 to 90 °C, holding for 5 min at each temperature increment. Negatively stained TEM images reveal that the fibers gradually disassemble as the temperature increases from 25 to 90 °C (Figure S2). Specifically, they remain intact below 40 °C and then gradually transition into undefined aggregates as the temperature increases above 50 °C (Figure S2). Circular dichroism (CD) spectroscopy was used to monitor peptide secondary structure during the fiber disassembly process (Figure S3). Assembled fibers exhibit a broad negative signal between ∼208 and 225 nm, which is consistent with our previous reports^18,19^ and can be attributed to a combination of PPII (polyproline II) and β-sheet secondary structure. With increasing temperature, the β-sheet signal (broad shoulder between 210 and 220 nm) gradually decreases in intensity, which indicates disruption of the β-sheet structure and is consistent with fiber disassembly (Figure S3a). We plotted the intensity of the CD signal at 208 and 220 nm versus temperature to monitor evolution of the secondary structure upon heating (Figure S3b). Signal intensity significantly decreases as the temperature increases above 40 °C, indicating the loss of β-sheet secondary structure (Figure S3b). We also conducted a ThT assay^41^ to monitor β-sheet structure within the fibers as a function of temperature (Figure S4). When excited at 450 nm, ThT strongly emits at 485 nm if it is associated with β-sheets in amyloid fibers;^41^ ThT emission is expected to decreases as β-sheets disassemble. C_18_-(PEP_Au_^M-ox^)_2_ fibers incubated with ThT show strong fluorescence at 485 nm from 25 to 35 °C (Figure S4). A dramatic decrease in ThT fluorescence is observed from 40 to 50 °C, indicating a significant loss in β-sheet secondary structure (Figure S4b). These results are consistent with our observations from TEM imaging and CD spectroscopy.

**Figure 3:**
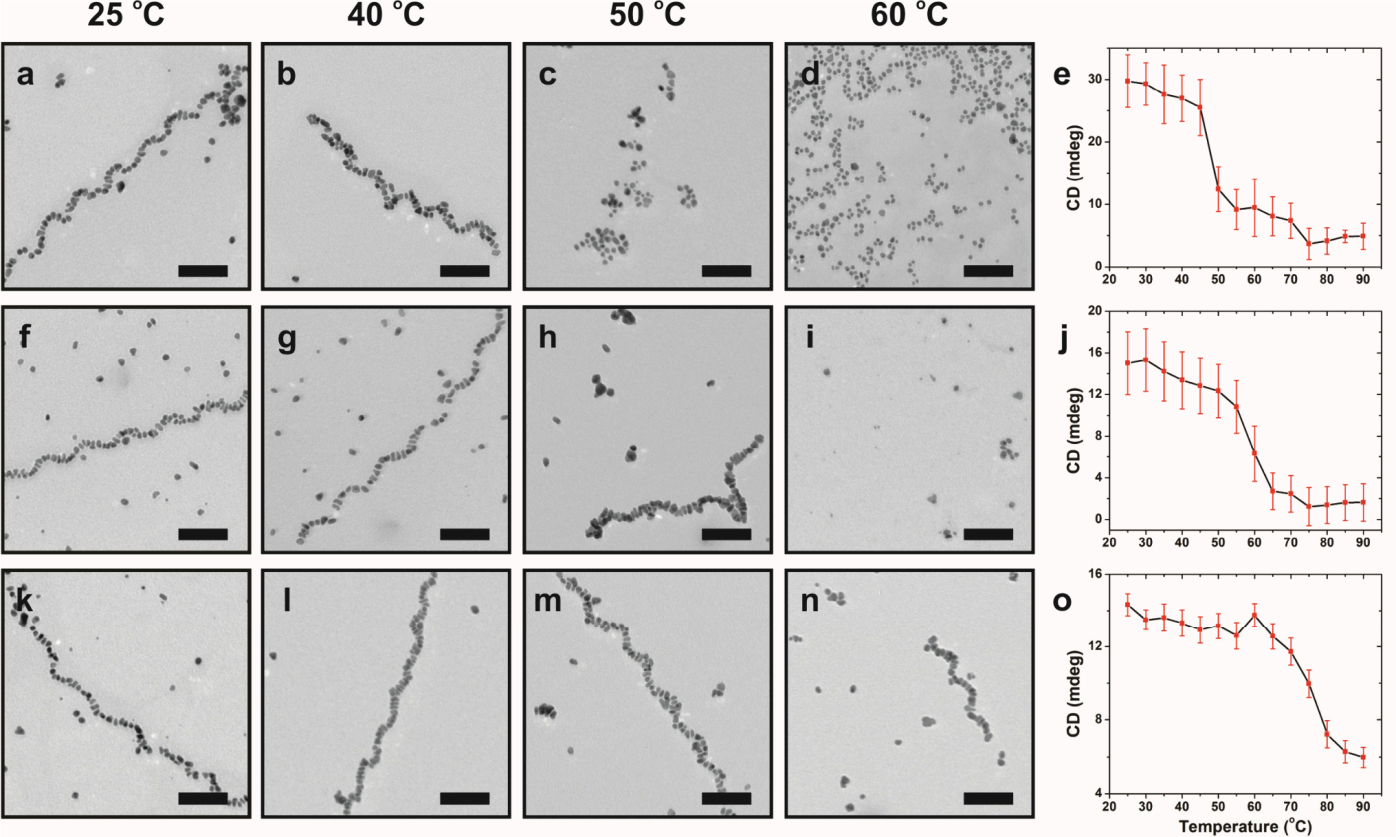
Thermal stability of **I**–**III**. TEM images (scale bar = 100 nm) and CD intensity at 560 nm for **I** (top row), **II** (middle row), and **III** (bottom row) at different temperatures. CD data points represent an average of three measurements, and the red bars represent standard deviation.

We next investigated the stability of the Au NP single-helix samples (**I**–**III**) as a function of temperature (Figure 3). In all three cases, the helices remained intact at low temperatures (below 40 °C) and disassembled at elevated temperatures (Figure 3). The temperature at which they begin to disassemble increases as NP dimensions increase. Based on TEM images, helices in **I**–**III** disassemble at ∼50 °C, ∼60 °C, and >60 °C, respectively (Figure 3c,i,n). Each sample exhibits a plasmonic chiroptical signal at ∼560 nm that derives from the chiral arrangement of the Au NPs; monitoring this signal as a function of temperature yields a melting profile we used to track helix disassembly (Figure 3e,j,g and Figure S5). For each sample, the temperature at which the plasmonic chiroptical signal steeply decreases correlates well with our TEM observations. A significant decrease in plasmonic chiroptical activity for **I** is observed from 40 to 50 °C (Figure 3e); **II** and **III** retain their plasmonic chiroptical activity until 50–60 °C (Figure 3j) and 60–70 °C (Figure 3o), respectively. Although all samples disassemble completely at temperatures greater than ∼80 °C, increasing the NP dimensions apparently increases thermal stability. We speculate that helix thermal stability increases with NP size because larger NPs will provide a bridge between more peptide conjugates within the fibers compared to smaller NP. In this scenario, both the NPs and intermolecular interactions serve to link the peptide conjugates together into fibers; the NPs may be viewed as an adhesive that helps prevent thermal denaturization of the peptide fibers.

### Chemical Denaturation Studies

In addition to thermal denaturation, it is well-known that various chemical denaturants can disrupt interpeptide interactions, which can lead to degradation of the peptide-based single helices. We chose to study the stability of **I**–**III** in the presence of urea, a representative denaturant used to disrupt the hydrogen-bonding interactions between assembled peptides.^42−44^ To determine an optimal amount of urea for these studies, we monitored C_18_-(PEP_Au_^M-ox^)_2_ fiber disassembly in the presence of different urea concentrations via both ThT assays (Figure S6) and TEM (Figure S7). Different amounts of urea were added to fiber samples in HEPES buffer, and the mixtures were allowed to sit for at least 24 h. The final concentrations for each component were as follows: 20 μM C_18_-(PEP_Au_^M-ox^)_2_, 0.1 M HEPES, 5 μM ThT, and 0.005–10 M urea. Samples treated with less than 2 M urea showed nonzero ThT emission, and ThT emission increased as [urea] decreased (Figure S6). Many intact fibers are observed via TEM for samples treated with 0.5 M urea (Figure S7a), which is consistent with the strong emission in the ThT assay (Figure S6). Increasing to 1 M urea results in a significant decrease in ThT emission, and TEM images reveal severe deformation and loss of fiber structure (Figures S6 and S7b). Finally, samples treated with 2 M urea or higher resulted in complete loss of ThT fluorescence, and no fibers were observed via TEM (Figures S6 and S7c). Based on these data, we elected to study helix stability at 0.5, 1, and 2 M urea.

**Figure 4:**
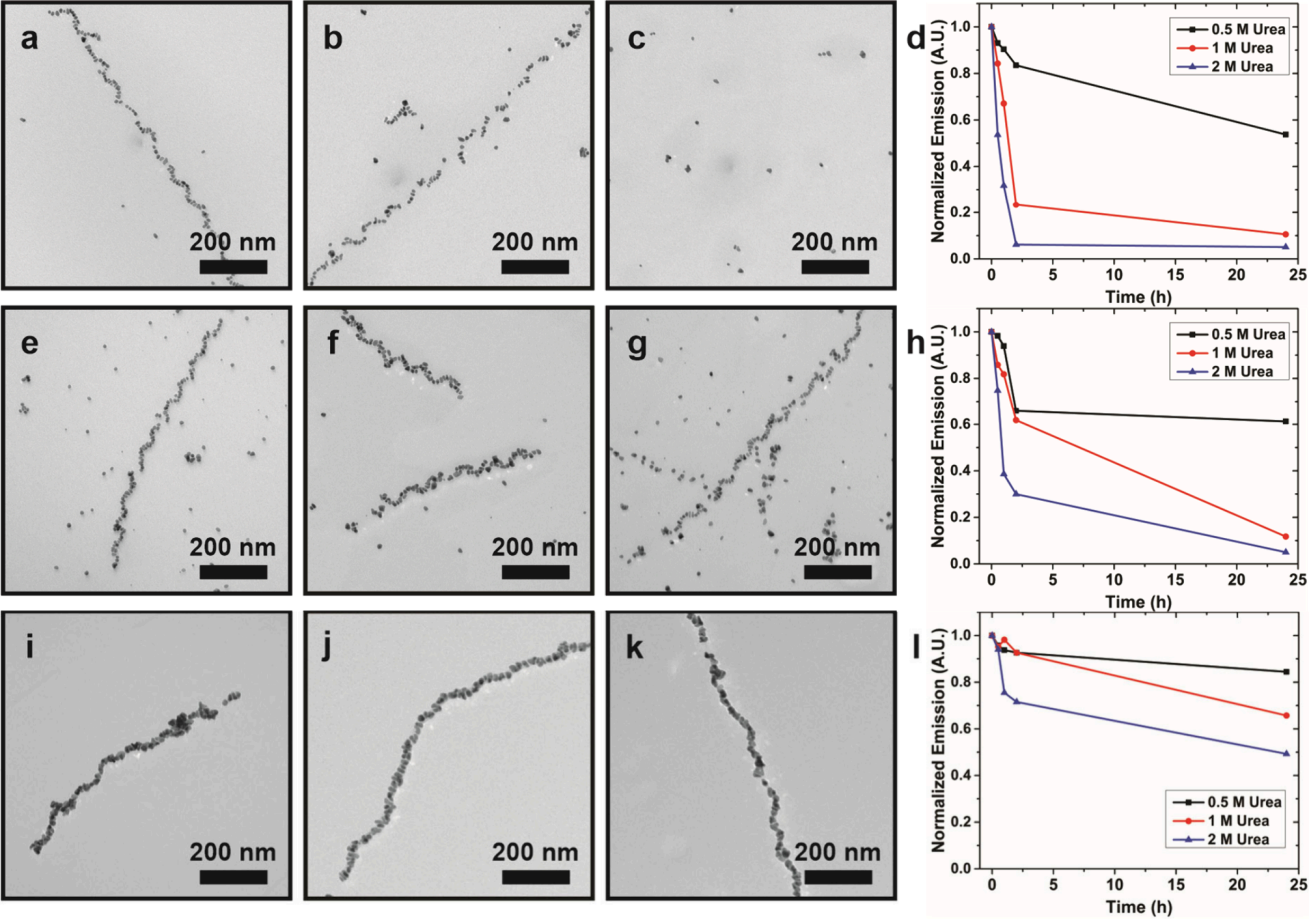
Affect of urea on **I**–**III** stability. TEM images of **I** (a, b, c), **II** (e, f, g), and **III** (i, j, k) after incubating in the presence of different urea concentrations (0.5 M: a, e, i; 1 M: b, f, j; 2 M: c, g, k) for 1 d. Normalized ThT emissions for **I** (d), **II** (h), and **III** (l) at 480 nm recorded for up to 1 d.

**I**–**III** exhibited different responses to urea. All helices remain largely intact in the presence of 0.5 M urea (Figure 4). Increasing to 2 M urea results in complete helix disassembly for **I** and significant helix fragmentation for **II** (Figure 4c,g). In contrast, helices in **III** remain intact at each urea concentration (Figure 4i,j,k). We also used ThT assays to further probe and understand the differential helix stabilities of **I**–**III** (Figure S8). For each sample, ThT emission decreased upon addition of urea, indicating denaturization of the peptide fibers (Figures 4d,h,l, S8). However, the magnitude of the decrease in ThT emission varied for **I**–**III** and with [urea]. For example, the ThT emission for **I** decreased significantly after 2 h of incubation with 2 M urea (Figures 4d, S8c), indicating fiber disassembly, which results in helix fragmentation (Figure 4c). The reduction in ThT emission becomes smaller as NP dimensions increase (Figure 4d,h,l), suggesting that the larger NPs afford some degree of protection against urea denaturization. To highlight this difference, after 24 h of incubation in 2 M urea, ThT emission for **III** is ∼50% of the initial intensity (Figure 4l), while ∼95% loss of ThT emission intensity is observed for **I** (Figure 4d). Again, we may attribute this improved stability to the postulate that, compared to smaller NPs, larger NPs will have a greater surface area to which peptides can bind; effectively, this means that larger NPs can bridge more peptides together than smaller NPs. If a denaturant such as urea disrupts the hydrogen bonding between the peptides in a fiber, a larger NP that bridges many peptides together within the fibers may help prevent fiber disassembly. In addition, the larger NPs may also prevent urea from accessing the peptide fiber.

### Peptide Digestion Studies

The chemical structure of C_18_-(PEP_Au_^M-ox^)_2_ remains intact during thermal and chemical denaturation. To determine the effect of peptide digestion on helix stability, we incubated **I**–**III** with ProK, a nonspecific broad-spectrum protease that is commonly used to digest peptides or proteins.^45,46^ In previous studies, we used ProK to digest peptide-based spherical Au NP superstructures.^47^ We first determined a suitable concentration of ProK for the digestion experiment. Lyophilized C_18_-(PEP_Au_^M-ox^)_2_ was dissolved in 0.1 M HEPES buffer and incubated for 30 min at 37 °C. At this temperature, C_18_-(PEP_Au_^M-ox^)_2_ fibers remain intact (*vide supra*) and ProK is active. We used both TEM imaging and ThT assays to monitor fiber assembly at 37 °C after incubation with ProK at concentrations ranging from 0.02 to 0.8 mg/mL (Figures S9 and S10). For these studies, the final concentrations for each component of the mixture were as follows: 20 μM C_18_-(PEP_Au_^M-ox^)_2_, 0.1 M HEPES, 5 μM ThT, and 0.02–0.8 mg/mL ProK. Results from ThT fluorescence and TEM imaging indicate that fibers remain intact for up to 18 h at the lowest ProK concentration (0.02 mg/mL) (Figure S9a and Figure S10a,b). At the higher ProK concentrations (0.1, 0.2, 0.4, and 0.8 mg/mL), the fibers degrade in less than 2 min, according to the ThT assays (Figure S9b–e). TEM images of these samples recorded after 30 min of incubation with ProK reveal globular aggregates and clear loss of fiber structure compared to the samples prior to ProK treatment (Figure S10c–j). Based on these screening studies, we elected to study single-helix stability at ProK concentrations of 0.02 and 0.4 mg/mL, expecting that the helices would remain intact at the lower concentration and disassemble at the higher concentration.

**Figure 5:**
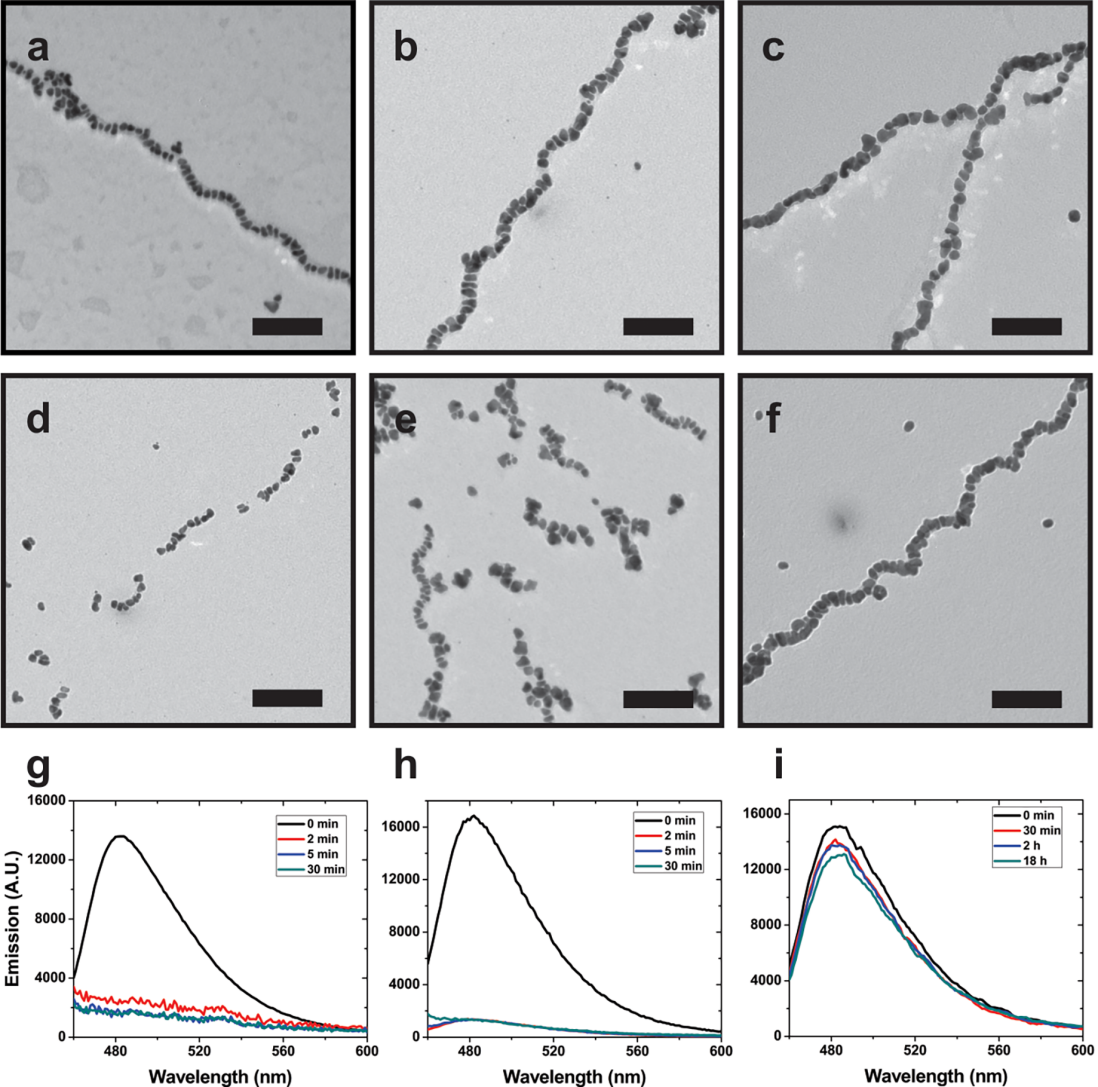
TEM images of **I**–**III** before (top row, a–c) and after (middle row, d–f) incubation with 0.4 M ProK for 30 min (scale bar = 100 nm). ThT fluorescence spectra of **I** (g), **II** (h), and **III** (i) were used to monitor digestion of peptide fibers.

**I**–**III** were incubated with 0.02 or 0.4 mg/mL ProK at 37 °C. At the lower ProK concentration (0.02 mg/mL), no obvious changes were observed in helix morphology for **I**–**III** after incubation for up to 18 h (Figure S11). After incubating with the higher ProK concentration (0.4 mg/mL) for 30 min (Figure 5), discrete NPs and fragmented single helices were observed for **I** and **II** (Figure 5d,e). Single helices in **III** remained intact after treatment with ProK (Figure 5f). We negatively stained TEM samples of **I**–**III** before and after incubation with ProK to visualize the peptide fibers (Figure S12). Prior to treatment with ProK (0.4 mg/mL), the peptide fiber is clearly visible as the scaffold around which the Au NPs are assembled (Figure S12a–c). After incubation with ProK (0.4 mg/mL), no fibers are observed in **I** and **II** (Figure S12d,e). For **III**, a faint fiber underlying the single helix can be detected (Figure S12f). ProK is commonly believed to target the C-terminus of peptides.^46^ Based on our established assembly model, Au NPs adhere to the C-terminal region.^18^ Less of the C-terminal region will be exposed when larger NPs are bound to the fibers. Therefore, larger NPs may provide more protection against ProK than smaller NPs. ThT fluorescence assays support this conclusion. For **I** and **II**, a dramatic decrease in ThT fluorescence occurs after 30 min of incubation with ProK, implying fiber digestion (Figure 5g,h); however, strong ThT fluorescence is maintained for **III** (Figure 5i), indicating that the C_18_-(PEP_Au_^M-ox^)_2_ fibers remain intact in this case.

### Colloidal Suspensions of Au NP Single Helices

**I**–**III** were prepared using syntheses that routinely yield single helices having lengths greater than 1 μm and heterogeneous length distributions.^21^ Consequently, the helices easily settle out of solution, which can potentially limit processability, use in applications that may require colloidal suspensions, and ease of incorporation into composite materials. Indeed, for a new material to gain more widespread application, it should not only be relatively stable and maintain its properties under stringent conditions but also be relatively homogeneous. Here, we combine what we previously learned about controlling the length of Au NP single helices^21^ with what we have described here about controlling their stability, to prepare thermally robust colloidal suspensions of short Au NP single helices that should ultimately be more easily processable and broadly applicable compared to heterogeneous **I**–**III**.

**Figure 6:**
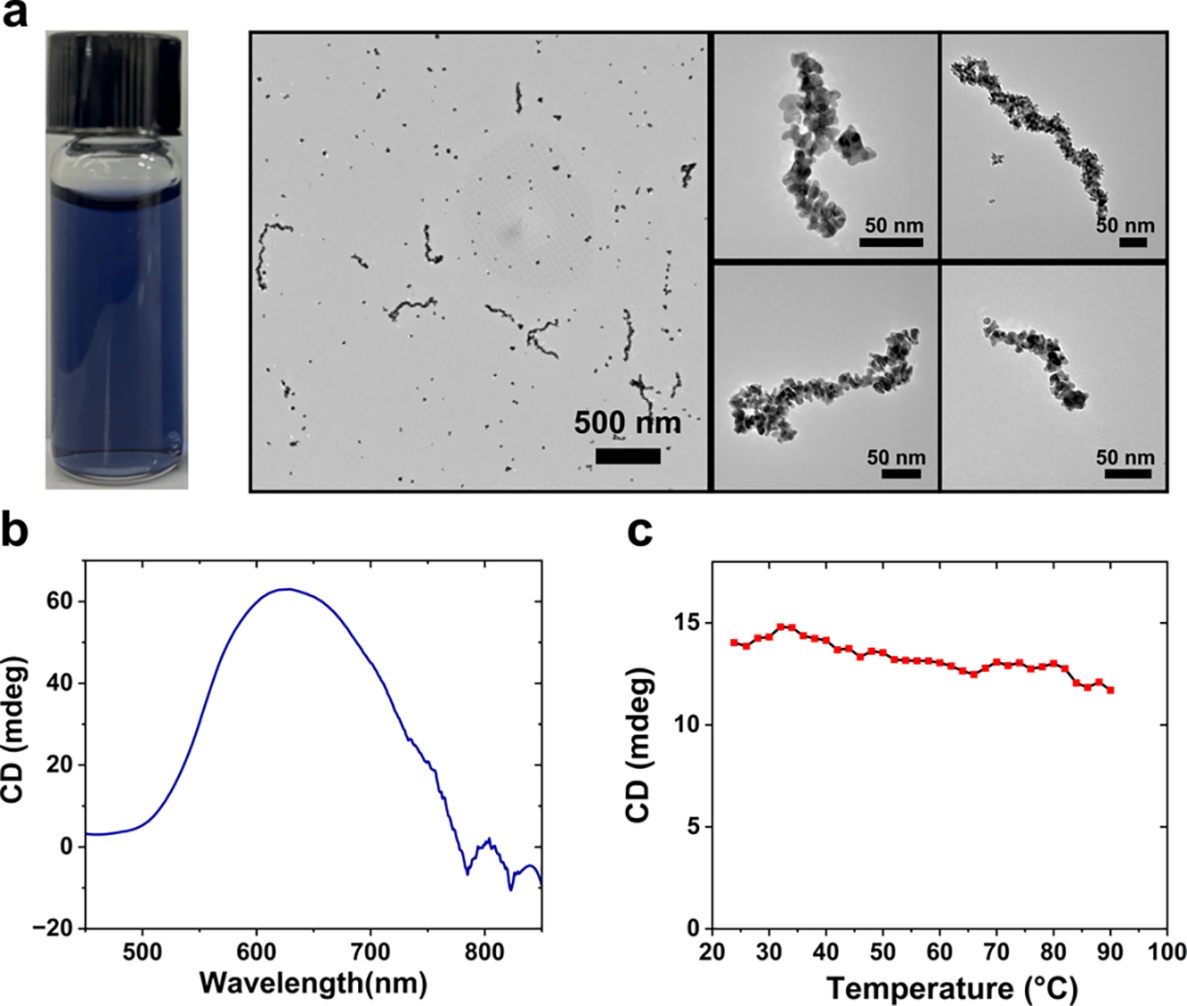
(a) Colloidal suspension of Au NP single helices and associated TEM images at different magnification. (b) Plasmonic CD spectrum of colloidal suspension. (c) Plasmonic CD spectrum monitored at 633 nm from 25 to 90 °C.

We recently reported a strategy for tuning the length of Au NP single helices using molecular peptide “modulators” that influence the kinetics of peptide assembly and fiber growth.^21^ While typical syntheses, such as those used to prepare samples **I**–**III**, yield a broad distribution of long (>1 μm) helices, adding a prescribed amount of modulator (nmol modulator ≥ nmol conjugate) allows for preparation of samples of short Au NP single helices having lengths of ∼200–400 nm and comparatively narrow length distributions.^21^ We modified our established synthesis for short Au NP single helices to create a dark blue colloidal suspension of short helices with larger constituent Au NPs (Figure 6). TEM images reveal the formation of short single helices (∼70% of helices are shorter than 300 nm; average length, ∼250 nm; median length ∼232 nm; data from ∼100 counts) comprising overlapping and interlinked irregularly shaped Au NPs (Figures 6a, S13). Importantly, the colloidal suspension exhibits strong plasmonic chiroptical activity (Figures 6b, S14), consistent with our previous reports.^18,19,21^ We examined the thermal stability of these helices by monitoring their plasmonic CD signal at 633 nm at 5 °C increments from 25 to 90 °C, with an incubation time of 5 min at each increment (Figure 6c). Notably, the plasmonic chiroptical signal was relatively stable until ∼80 °C, decaying only ∼14% compared to the initial measurement at 25 °C. This result underlines the structural robustness of these helical colloids. TEM images collected at various temperatures all show the presence of intact short helices (Figure S15), consistent with observation of strong plasmonic chiroptical response.

## Conclusion

We examined the structural stability of Au NP single helices as a function of particle dimensions at elevated temperatures, in the presence of a chemical denaturant (urea), and in the presence of a peptide digestion agent (ProK), conditions that are relevant to the underlying peptide template. Our data indicate that helices with larger NPs are more structurally robust compared to helices with smaller NPs. We reason that larger NPs impart greater stability to the underlying peptide scaffold, making them more resistant to denaturation due to either heat or chemical denaturants. In the case of treatment with ProK, we speculate that the larger NPs may shield the underlying peptide fiber from digestion. Finally, we demonstrated that short single helices with large, intergrown constituent NPs can be prepared as a thermally stable colloidal suspension that maintains strong plasmonic chiroptical activity after heating up to ∼80 °C. These results significantly expand our understanding of the stability of these materials, and the studies represent a significant step forward toward their broader use and application.

## Supporting Information Available

Additional chemical structures, CD data, UV-vis spectra, ThT assay spectra, and TEM images (PDF)
