# Supplementary material for: Imparting Stability to Chiral Helical Gold Nanoparticle Superstructures
Source: Langmuir. 2024 Jul 17;40(30):15700–9. doi: 10.1021/acs.langmuir.4c01531 (PMC11295200; doi:10.1021/acs.langmuir.4c01531)
Supplement: Supplementary file 1 — la4c01531_si_001.pdf [file la4c01531_si_001.pdf]

Supporting Information

Imparting Stability to Chiral Helical Gold Nanoparticle Superstructures

Yicheng Zhou[†,§], Yuyu Zhang[†,§], Nathaniel L. Rosi*[†‡]

† Department of Chemistry, University of Pittsburgh, Pittsburgh, Pennsylvania 15260, United States
‡ Department of Chemical and Petroleum Engineering, University of Pittsburgh, Pennsylvania 15260, United States
§ These authors contributed equally

Table of Contents

1. Synthesis and Structure of Peptide Conjugates 2
1.1 Synthetic scheme for the preparation of divalent peptide conjugates 2
1.2 Molecular structure 3
2. Supporting Data 4

1. Synthesis and Structure of Peptide Conjugates

1.1 Synthetic scheme for the preparation of divalent peptide conjugates

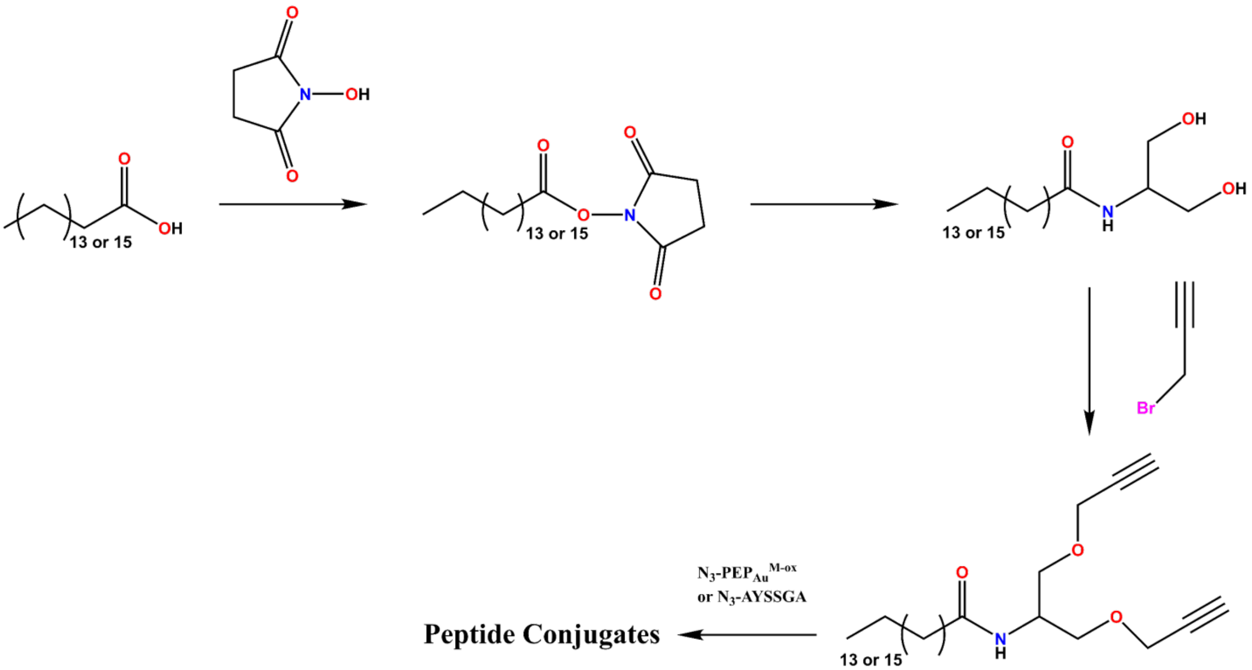

1.2 Molecular structure

a C_{18}-$(PEP_{Au}^{M\text{-}ox})_2$

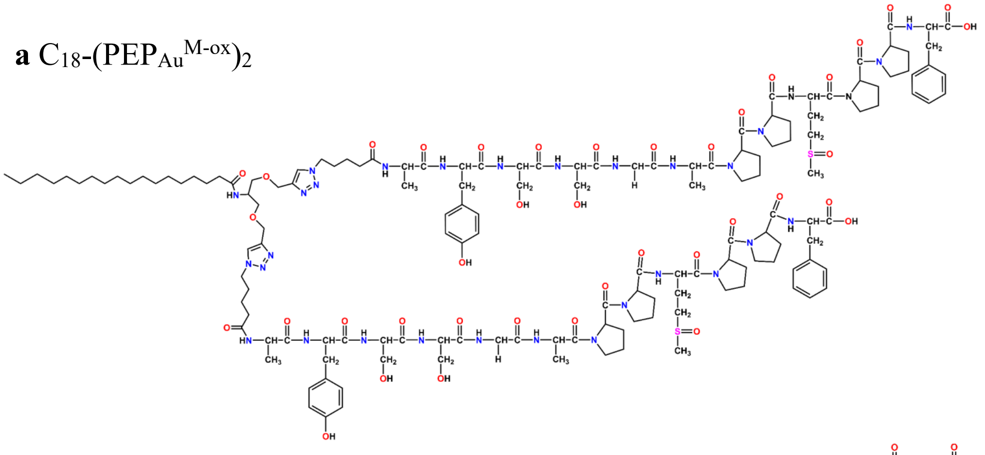

b C_{16}-$(PEP_{Au}^{M\text{-}ox})_2$

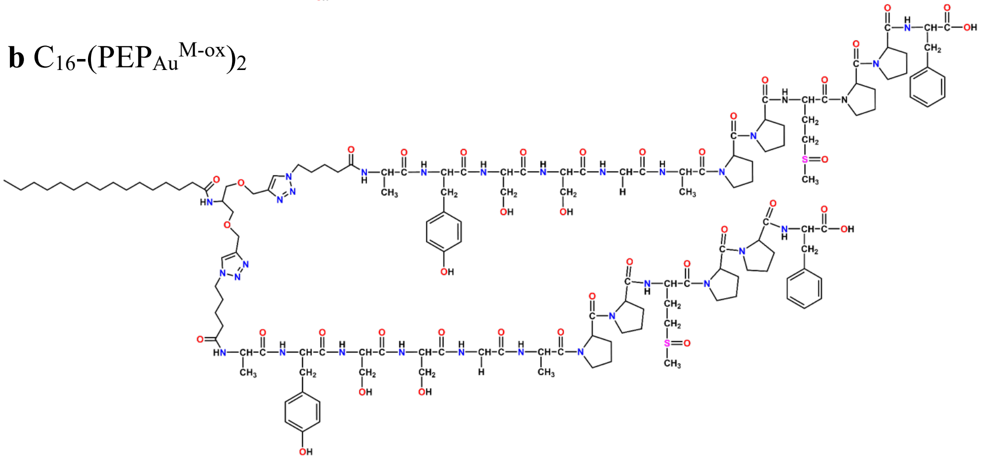

c C_{16}-$(AYSSGA)_2$

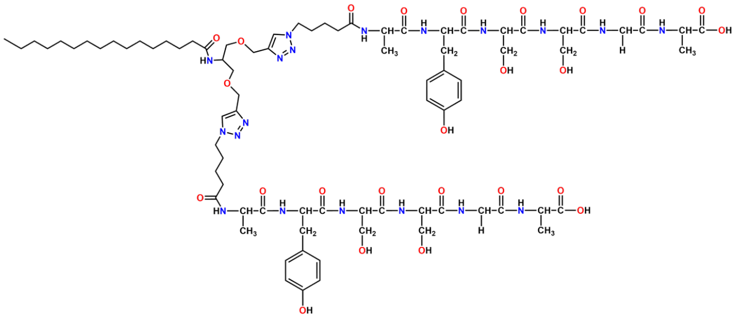

2. Supporting Data

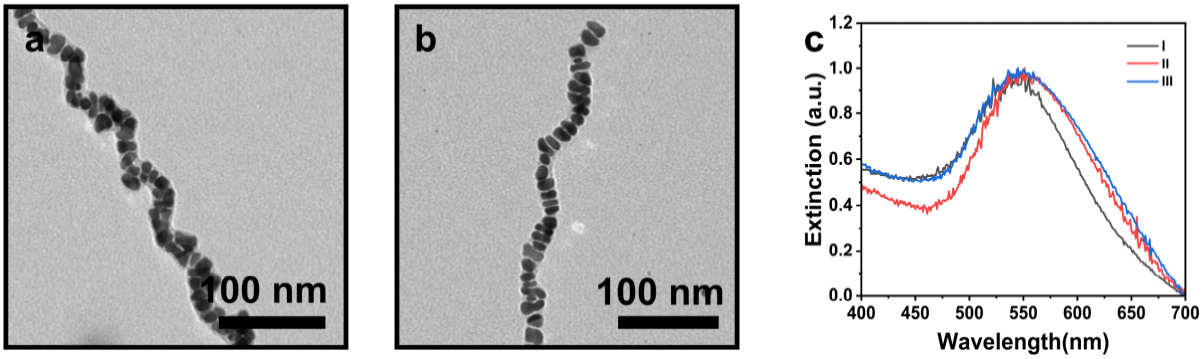

Figure S1. Zoomed-in TEM images of a) **II** and b) **III**. c) Normalized UV-Vis extinction spectrum of **I-III**.

Thermal Stability Studies

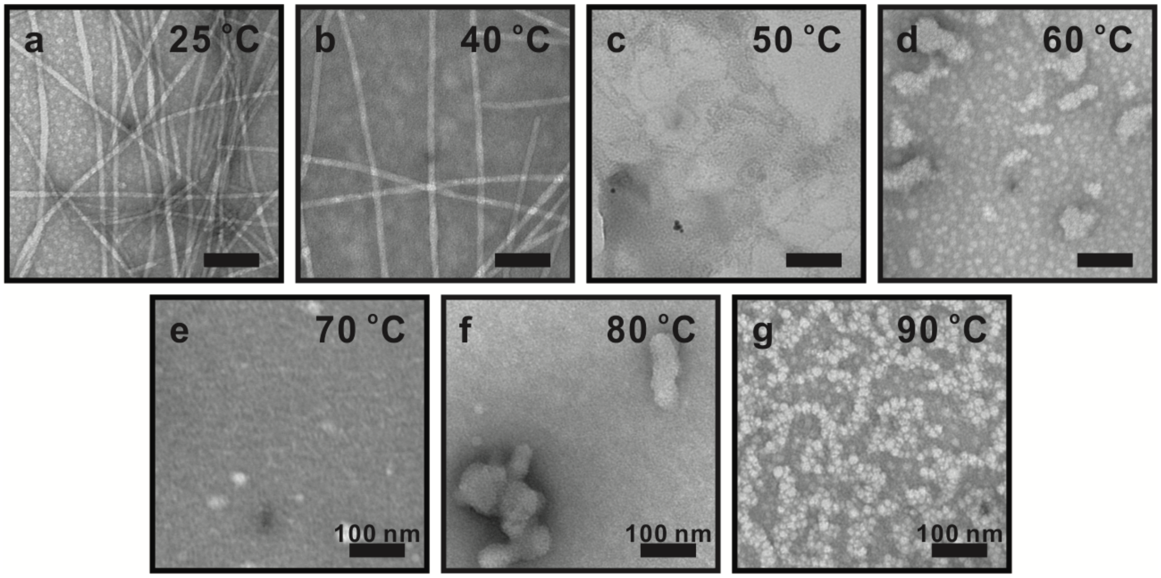

Figure S2. Negatively-stained TEM images of C_{18}-($PEP_{Au}^{M\text{-}ox}$)$_2$ assemblies in 0.1 M HEPES at different temperatures.

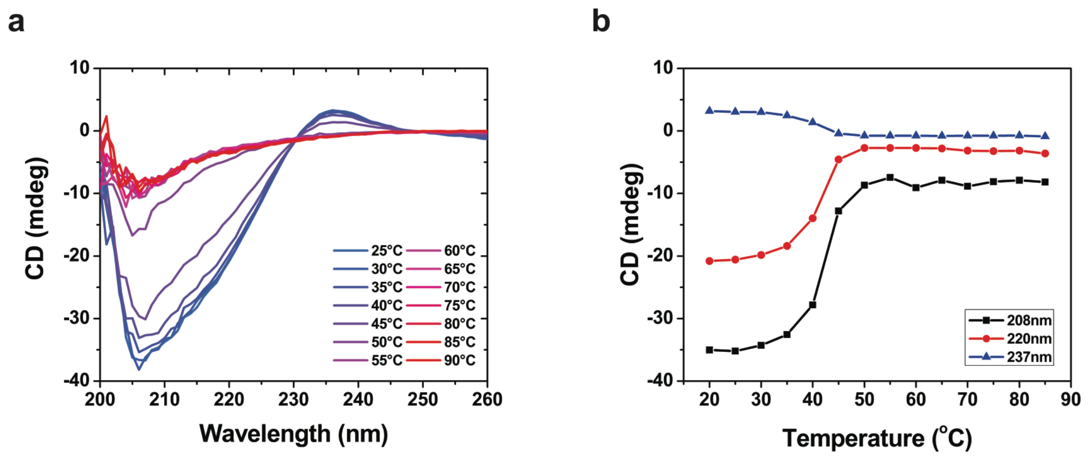

Figure S3. (a) CD spectra of C_{18}-$(PEP_{Au}^{M\text{-}ox})_2$-based fibers at different temperatures; (b) CD signal of C_{18}-$(PEP_{Au}^{M\text{-}ox})_2$-based fibers at 208 nm (black), 220 nm (red), and 237 nm (blue) at different temperatures.

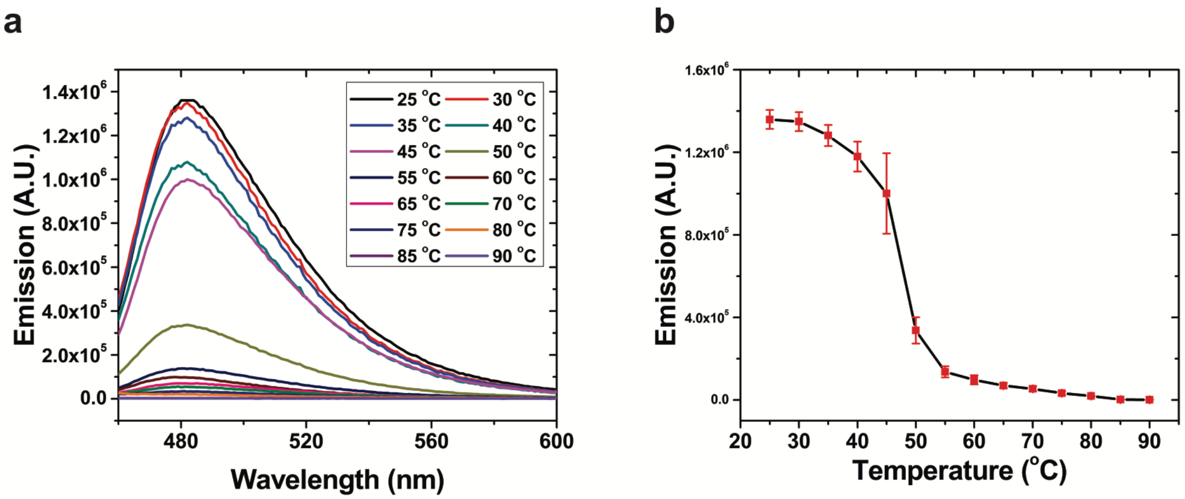

Figure S4. (a) ThT fluorescence spectra of C_{18}-$(PEP_{Au}^{M\text{-}ox})_2$-based fibers at different temperatures (b) ThT fluorescence emission signal at 485 nm of C_{18}-$(PEP_{Au}^{M\text{-}ox})_2$-based fibers at different temperatures.

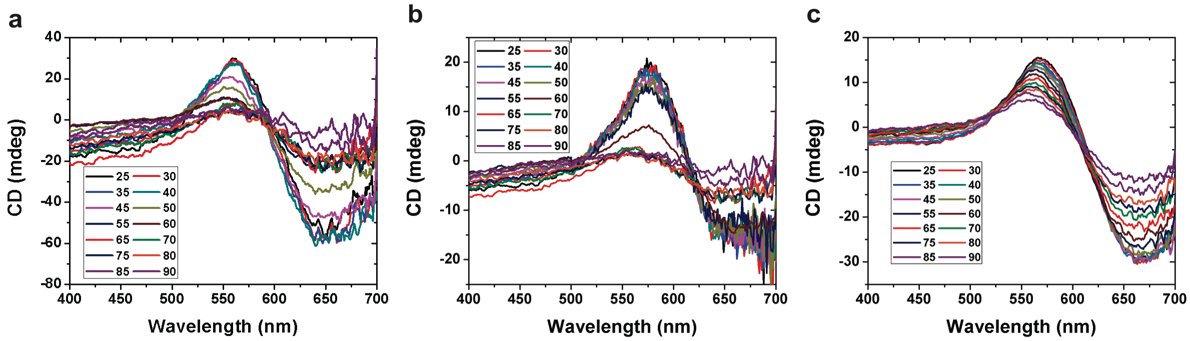

Figure S5. CD spectra of **I** (a), **II** (b), and **III** (c) Au NP single helices at different temperatures (in °C).

Stability Studies in Presence of Urea

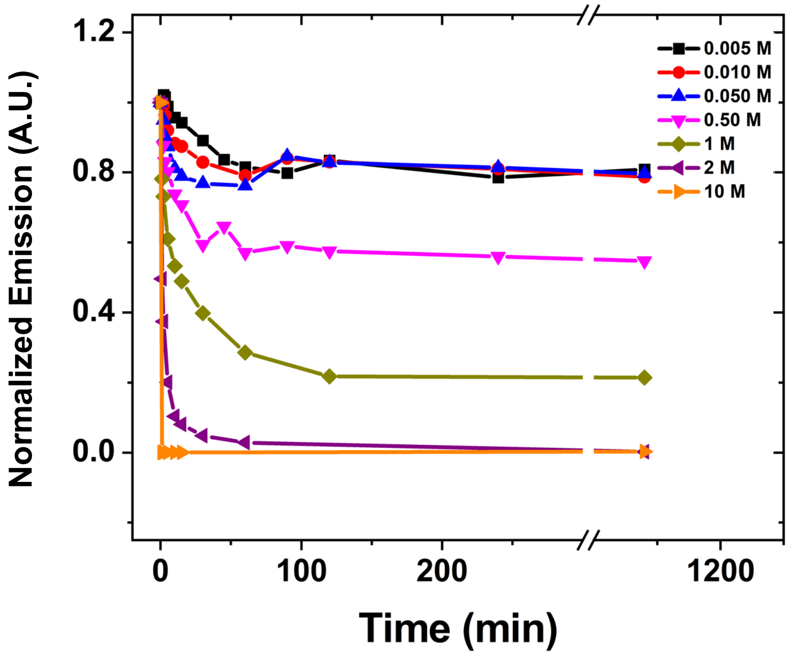

Figure S6. Normalized ThT fluorescence monitored at 485 nm of C_{18}-$(PEP_{Au}^{M\text{-}ox})_2$-based fibers in the presence of varying concentrations of urea (0.005 M-10 M).

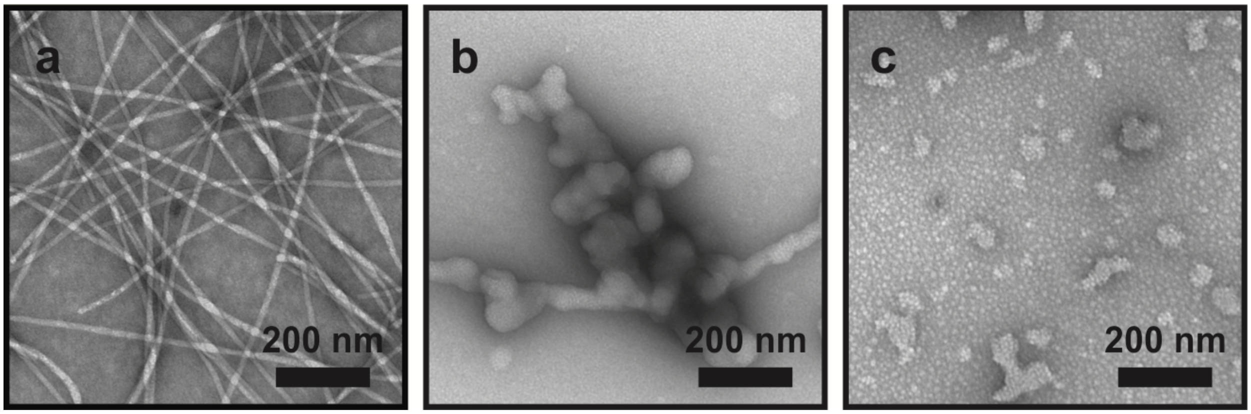

Figure S7. Representative negatively-stained TEM images of C_{18}-$(PEP_{Au}^{M\text{-}ox})_2$-based fibers after incubating in the presence of varying concentrations of urea (a) 0.5 M, (b) 1 M, and (c) 2 M.

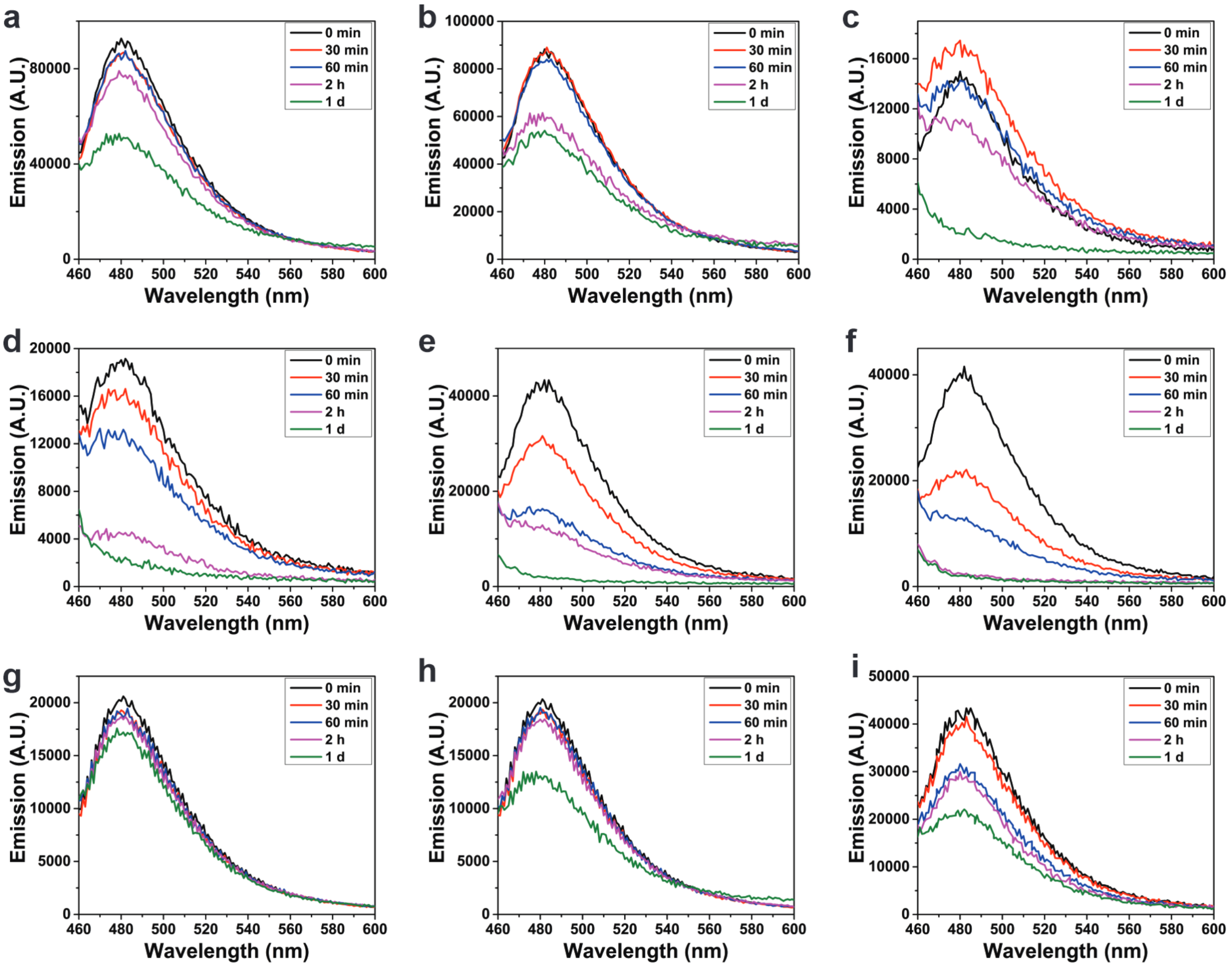

Figure S8. ThT fluorescence spectra of **I** (a, b, c), **II** (d, e, f), and **III** (g, h, i) upon incubating in the presence of varying urea concentrations at 0.5 M (a, d, g), 1 M (b, e, h), 2 M (c, f, i).

Stability Studies in Presence of Proteinase K

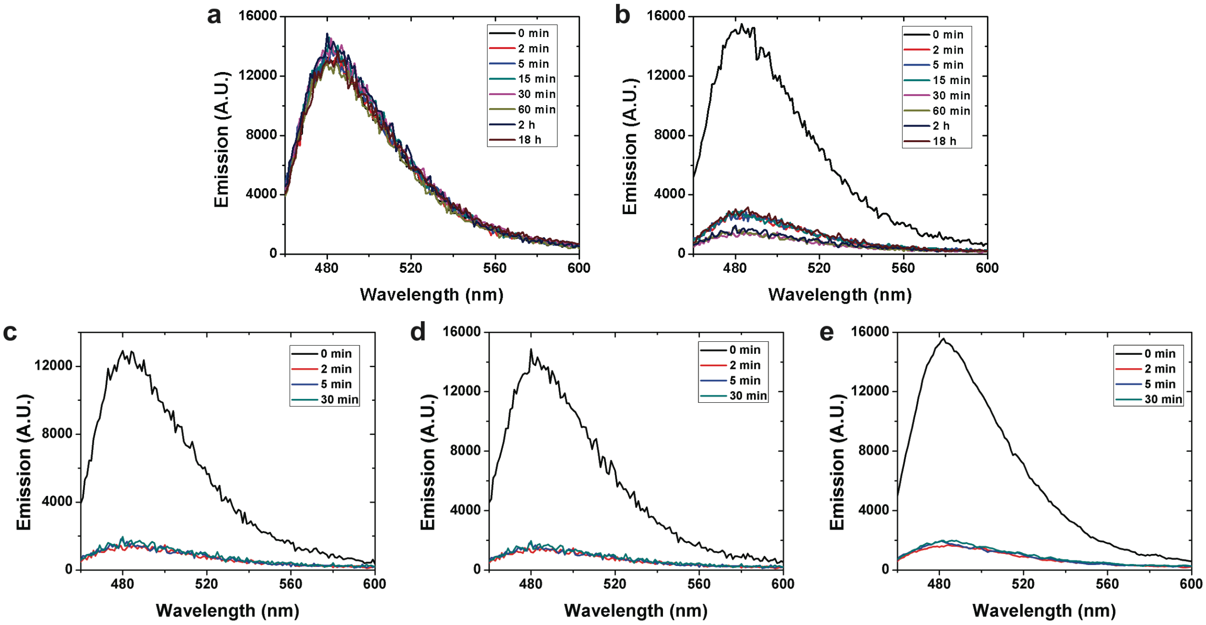

Figure S9. ThT fluorescence spectrum of C_{18}-($PEP_{Au}^{M\text{-}ox}$)$_2$-based fibers in the presence of different concentrations of ProK: (a) 0.02 mg/mL, (b) 0.1 mg/mL, (c) 0.2 mg/mL, (d) 0.4 mg/mL, (e) 0.8 mg/mL at 37 °C.

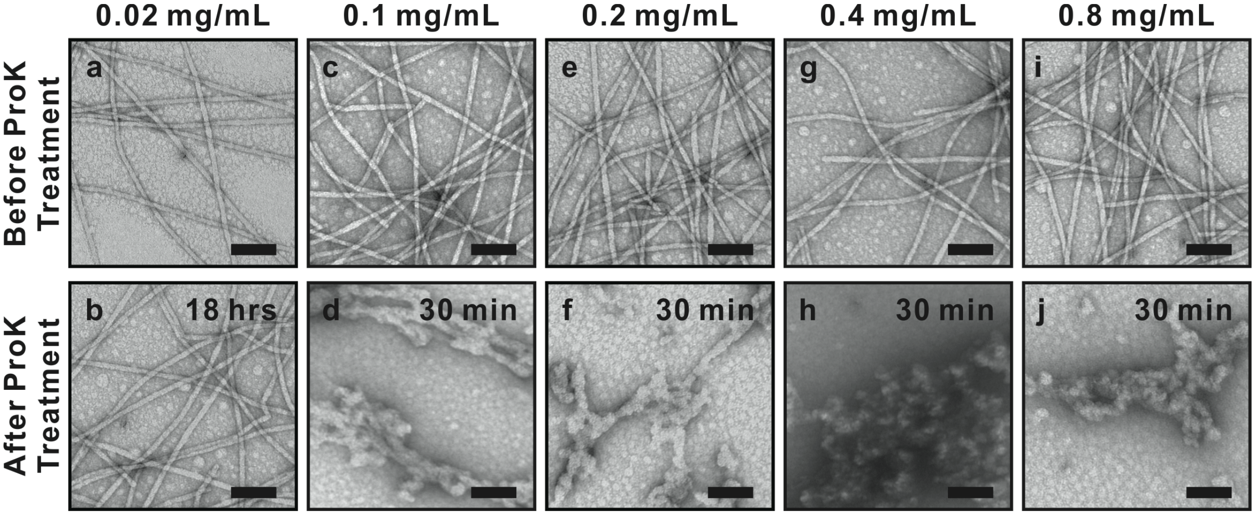

Figure S10. Representative negatively-stained TEM images of C_{18}-($PEP_{Au}^{M\text{-}ox}$)$_2$-based fibers before (top row) and after (bottom row) incubation with different concentrations of ProK: (a, b) 0.02 mg/mL, (c, d) 0.1 mg/mL, (e, f) 0.2 mg/mL, (g, h) 0.4 mg/mL, (i, j) 0.8 mg/mL. For 0.02 mg/mL ProK, the TEM image was collected after 18 h of incubation; for all other ProK concentrations, TEM images were collected after 30 min of incubation. Scale bar = 100 nm.

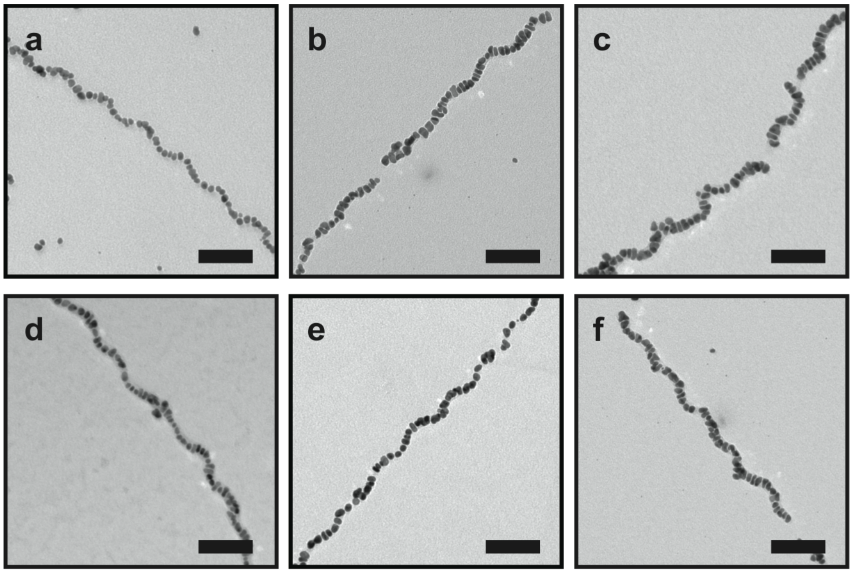

Figure S11. Representative TEM images of **I** (a, d), **II** (b, e), and **III** (c, f) before (top row) and after (bottom row) incubating with 0.02 mg/mL ProK for 18 h. Scale bar = 100 nm.

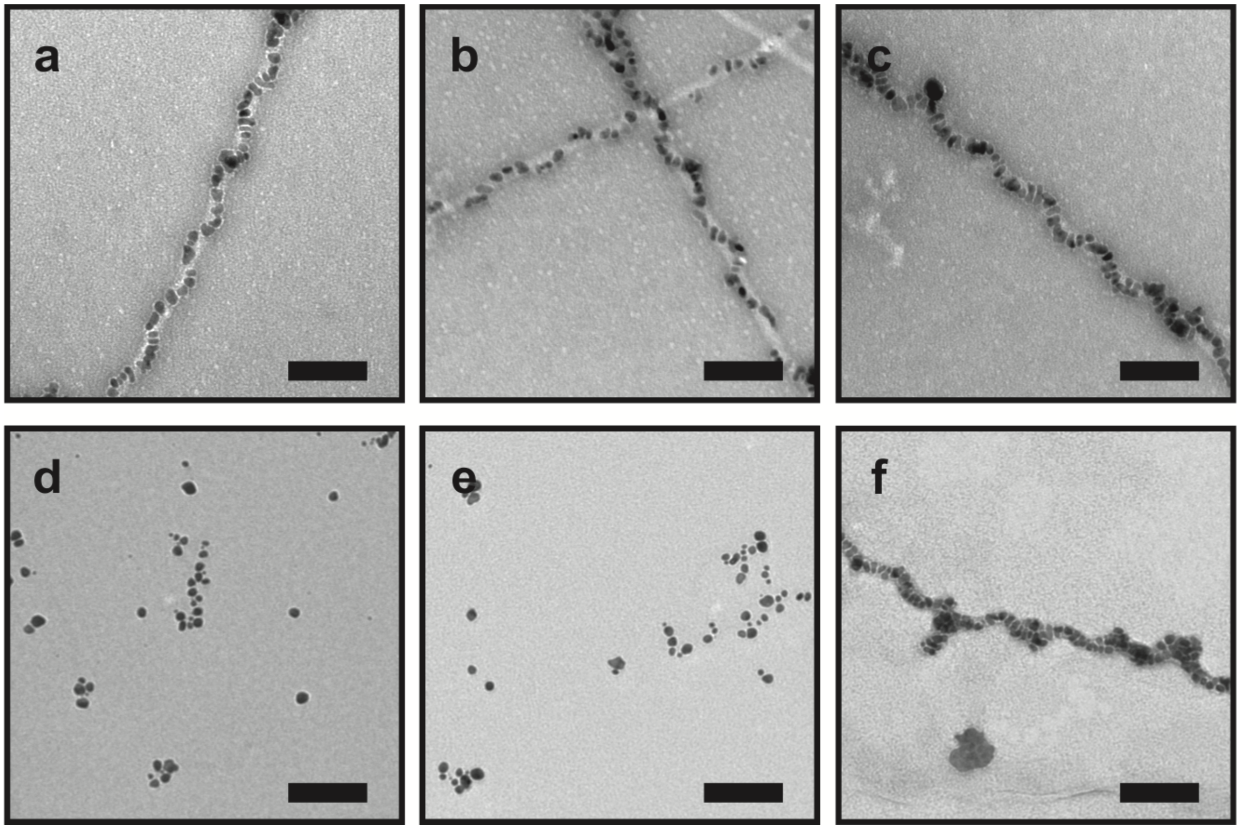

Figure S12. Representative negatively-stained TEM images of **I** (a, d), **II** (b, e), and **III** (c, f) before (top row) and after (bottom row) incubating with 0.4 mg/mL ProK for 30 min. Scale bar = 100 nm.

Colloidal Suspensions of Single Helices

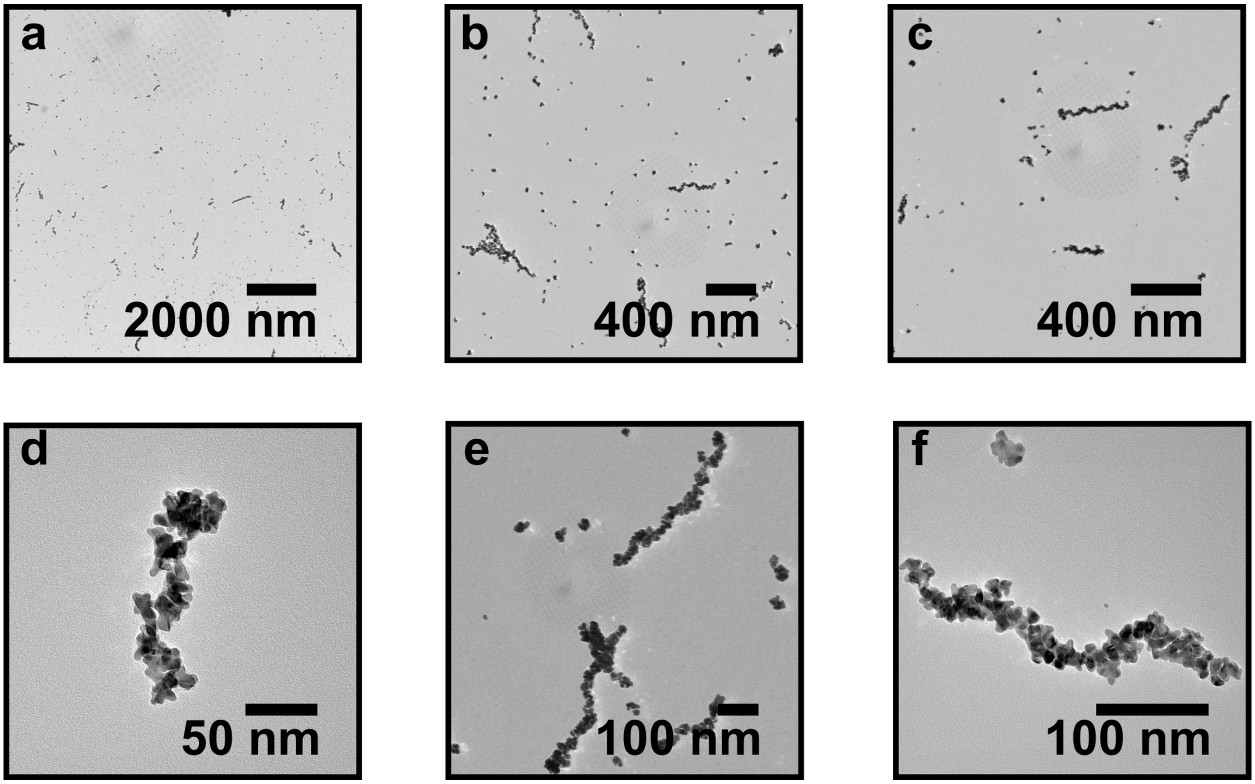

Figure S13. Additional TEM images of single helices colloidal suspension.

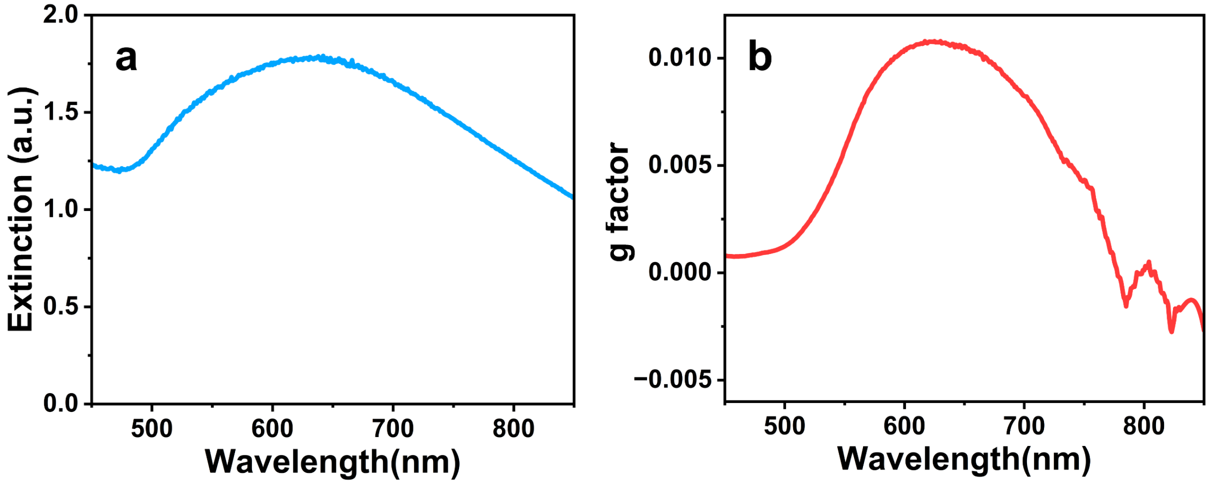

Figure S14. Plasmonic chiroptical activity of single helices colloidal suspension. (a) UV-Vis spectrum and (c) anisotropy factor (g) plot, determined by dividing molar circular dichroism by the molar extinction.

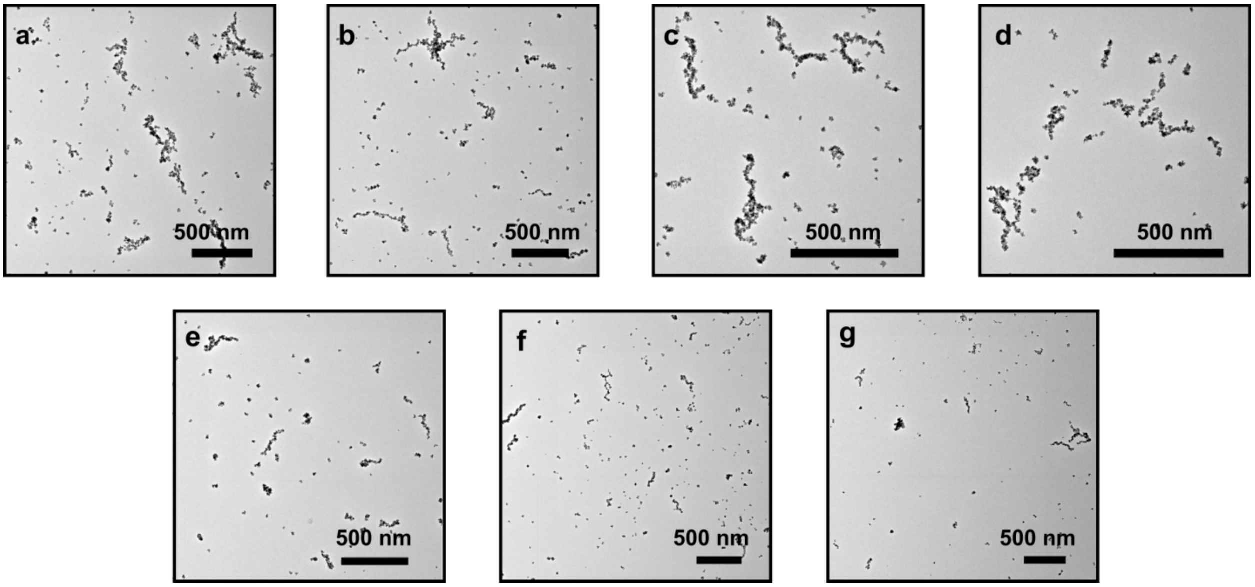

Figure S15. Representative TEM images of single helices colloidal suspension at varying temperatures: (a) 25 °C, (b) 40 °C, (c) 50 °C, (d) 60 °C, (e) 70 °C, (f) 80 °C, to (g) 90 °C.
